# Early Alterations in Glucose Homeostasis Associated with a Family History of Diabetes Mellitus: A Systematic Review and Meta-Analysis

**DOI:** 10.3390/medsci14030349

**Published:** 2026-06-26

**Authors:** Karen Dennise Lozada Tobar, Laura Cristina Nonato, Leticia Nunes Dilelli, Alexandre Konig Garcia Prado, Ana Carolina Ghezzi, Lucieli Teresa Cambri

**Affiliations:** 1 Postgraduate Program in Physical Education, Federal University of Mato Grosso, Cuiabá 78060-900, MT, Brazilleticiadilellibio@gmail.com (L.N.D.); akgprado@gmail.com (A.K.G.P.); 2 Postgraduate Program in Health Sciences, Federal University of Mato Grosso, Cuiabá 78060-900, MT, Brazil; 3 School of Medicine and Health Science, The George Washington University, Washington 20052, DC, USA; carolgehzzi@gmail.com

**Keywords:** first-degree relative, glycemia, heritability, insulin, parental diabetes, risk of diabetes

## Abstract

Background and Aims: To evaluate whether the family history of diabetes mellitus (FHD^+^) is associated with markers of glucose homeostasis in healthy adults. Methods: Studies evaluating adults aged 18 to 60 years without a diagnosis of cardiometabolic disease, and reporting the influence of FHD^+^ (at least one first-degree relative) on fasting and 2 h postload glucose, and fasting insulin were included. The electronic database MEDLINE (via PubMed) was searched in February 2026 for studies published in English. Results are presented as mean differences with 95% confidence intervals, using random-effects models. Sensitivity analyses were performed considering study design, methodological quality, the definition of FHD^+^, and participants’ age and sex. Results: Twenty-six studies totaling 3122 individuals were included. Fasting glucose [3.48 (1.34–5.63) mg·dL^−1^; – = 90%], 2 h postload glucose [4.18 (2.27–6.10) mg·dL^−1^; I^2^ = 38%], A1c [0.12 (0.04–0.19)%; – = 67%]; fasting insulin [1.72 (0.97–2.48) µU·mL^−1^; – = 90%], and HOMA–IR [0.55 (0.42–0.69); – = 70%] were higher (*p* < 0.001) in individuals with an FHD^+^. Meta-regression analyses showed no significant associations between mean age or BMI and markers of glucose homeostasis. Findings remained robust across sensitivity and subgroup analyses, with reduced heterogeneity for some outcomes. Conclusions: The available evidence suggests that FHD^+^ may be associated with markers of impaired glucose homeostasis in healthy adults. However, these results should be interpreted with caution and confirmed in higher-quality prospective studies.

## 1. Introduction

Type 2 diabetes mellitus (T2DM) is a chronic metabolic disease and a leading cause of morbidity and mortality, primarily due to its association with cardiovascular complications. T2DM represents a major global public health issue, accounting for approximately 90% of all diagnosed cases. Recent estimates from the International Diabetes Federation reported that the global prevalence of diabetes was 11.1% (~589 million) in 2024, projected to rise to 13.0% (~853 million) by 2050, with an increasing impact on younger populations, further amplifying its long-term health and socioeconomic consequences [1].

Family history is a well-established non-modifiable risk factor for various chronic noncommunicable diseases. It represents an overall construct encompassing multiple factors, including, but not limited to, diet, physical activity, socioeconomic factors, and genetic susceptibility. Those with a positive family history of diabetes (FHD^+^) have a 2- to 3.5-fold higher risk for developing diabetes compared to those without such a history [2,3], while the risk rises to 6.0-fold in individuals with two diabetic parents. FHD^+^ seems associated with an earlier age of onset and poorer glycemic control. In a study from 2017, among the participants whose age of onset of diabetes was between 21 and 30 years, 91% had FHD^+^ [4].

Previous studies have suggested that impairments in glucose homeostasis may be associated with alterations in autonomic function even before T2DM clinically manifests [5]. However, evidence regarding autonomic dysfunction, including heart rate variability (HRV), in healthy individuals with a FHD^+^ remains limited [6].

Family history can guide preventive strategies and facilitate the early identification and management of chronic diseases, such as T2DM. Therefore, family history may be a practical and accessible tool to identify individuals at increased risk of developing T2DM and support the early implementation of preventive strategies, with behavioral modifications, including nutritional interventions, that could potentially delay disease onset and improve health outcomes. Although numerous studies have demonstrated that FHD^+^ can impair the metabolic outcomes of offspring, robust evidence regarding its impact on adults remains sparse and contested, with some studies showing high fasting [7,8,9,10,11,12,13,14] and/or 2 h postload glucose [6,8,15], and insulin [7,8,11,12,16,17,18] and no influence of FHD^+^ [18,19,20,21,22,23,24,25,26,27,28,29,30,31]. However, the magnitude and consistency of these associations across studies remain unclear. Limited statistical power and lack of control for confounding factors (i.e., age, sex, and BMI)) [7,10,11,13,16,18,19,26,29,31] may partly explain these divergent findings. In addition, outcomes are often assessed in isolation, which may obscure the broader metabolic pattern associated with FHD^+^. These inconsistencies limit clinical translation and highlight the need for a systematic quantitative synthesis of the relationship between family history and early alterations in glucose homeostasis. To our knowledge, no previous meta-analysis has comprehensively synthesized the evidence on the association between FHD^+^ and markers of glucose homeostasis in adults. This meta-analysis aimed to evaluate whether FHD^+^ is associated with markers of glucose homeostasis in adults, including fasting glucose, 2 h postload glucose, glycated hemoglobin (A1c), fasting insulin, and HOMA–IR.

## 2. Methods

### 2.1. Search Strategy

The study protocol was registered in the International Prospective Register of Systematic Reviews (PROSPERO) under the number CRD420251107652 and followed PRISMA guidelines [32]. To identify eligible studies for inclusion in the meta-analysis, a literature search was conducted in December 2024 and updated on 16 February 2026 using the electronic database MEDLINE (via PubMed), covering records from 1 January 2004. The search strategy included the terms in the title/abstract, with Boolean operators: ((“family history of diabetes” OR “diabetes family history” OR “genetic predisposition” OR “parental diabetes” OR “diabetes inheritance” OR “hereditary diabetes” OR “diabetes risk”) AND (“glucose” OR “blood sugar” OR “glycemic response” OR “glycemia” OR “glycaemic control” OR “hyperglycemia” OR “glycemic variability” OR “glycemic control” OR “glycated hemoglobin” OR “HbA1c” OR “glycohemoglobin” OR “A1c” OR “glycosylated hemoglobin” OR “hemoglobin A1c” OR “glyco-Hb” OR “glycated haemoglobin” OR “glycosylated haemoglobin” OR “insulin” OR “heart rate variability” OR “cardiac autonomic modulation” OR “autonomic modulation” OR “cardiac autonomic dysfunction” OR “autonomic dysfunction” OR “autonomic function” OR “autonomic nervous system” OR “sympathetic system” OR “sympathetic modulation” OR “parasympathetic system” OR “parasympathetic modulation” OR “autonomic responses”)).

### 2.2. Study Eligibility Criteria

The following eligibility criteria (Table S1—Supplementary Material), taken from the PICOS (Population, Intervention, Comparator, Outcomes, and Study) framework were considered for inclusion of studies in this meta-analysis: (a) studies that included adults of both sexes, aged between 18 and 60 years, who had no diagnosed cardiometabolic conditions (obesity, type 2 diabetes, hypertension) or additional risk factors, such as dyslipidemia; (b) study design that was within the FH context, FHD^+^, considering first-degree relative at least one first-degree relatives (parents and/or siblings) as FHD; (c) studies with at least one primary outcome measure that were fasting glucose and 2 h postload glucose, glycated hemoglobin (A1c), insulin, HOMA-IR and HRV indices; (d) studies that had secondary outcome measures of body mass index (BMI) and blood pressure (BP); (e) studies with the full text published in English; and (f) studies that included observational (cross-sectional and prospective study) and interventional (baseline data). We did not include studies that were: (a) non-primary studies; (b) animal studies; (c) studies including children (<18 years) or older adults (>60 years); (d) studies that did not include a control group (i.e., FHD^−^); (e) studies in which FHD^+^ was defined based on second-degree relatives (i.e., grandparents); (f) studies that did not clearly specify the degree of family relationship used to define FHD^+^; (g) studies not presenting numeric data required for the meta-analysis. In this case, we contacted the corresponding authors to obtain data not available in the full text, but when no response was received, these data were not included. The clinical variables extracted were fasting glucose and/or 2 h postload glucose (expressed in mg·dL^−1^ or mmol·L^−1^), A1c (%), fasting insulin (expressed in µU·mL^−1^, pmol·L^−1^), HOMA–IR, systolic and diastolic BP (expressed in mmHg), and BMI (expressed in kg·m^−2^). Only one eligible study evaluated HRV; therefore, a quantitative synthesis (meta-analysis) could not be performed for this outcome.

### 2.3. Data Extraction

Initially, studies were searched for and imported into the Rayyan web application (https://rayyan.qcri.org). Three reviewers (KDLT, LGD, and LCN) independently assessed the titles and abstracts retrieved from the electronic database to identify articles eligible for the full-text assessment, with all cases of disagreement discussed until consensus was reached. When abstracts did not provide enough information to assess eligibility, the corresponding full-text articles were retrieved for evaluation. Reviewers (KDLT, LGD, and LCN) independently assessed the full-text articles to determine whether the studies met the eligibility criteria. A fourth reviewer (LTC.) was consulted to resolve disagreements regarding inclusion/exclusion. The data extraction (author and year, participants, mean, and standard deviation—SD of outcomes) was also made independently (LGD and LCN) and further compared. We considered results reported separately for individuals with just one diabetic parent and those with both parents being diabetic [7], as independent studies.

### 2.4. Statistical Analyses

Results are presented as absolute mean difference between groups with a 95% confidence interval, since outcomes in all studies were reported on the same scale. Some data were presented in different units (mmoL·L^−1^ and pmoL·L^−1^), such as glucose and insulin data, which we converted to mg·dL^−1^ or µU·mL^−1^. When data were presented as the standard error of the mean (SEM), we calculated the SD (=SEM √n). The risk of bias was assessed independently by two reviewers (ACG and LTC) using an adapted version of the Newcastle–Ottawa Scale (NOS) for observational studies [33], evaluating selection (four items), comparability (one item), and outcome domains (two items), with a maximum score of ten stars (higher scores indicating better study quality). The scale ranges from 0 to 10 points: 0–4 indicates unsatisfactory quality, 5–6 satisfactory, 7–8 good, and 9–10 very good. Discrepancies were resolved by consensus. Statistical heterogeneity across studies was examined using Cochran’s Q test and quantified with the I^2^ statistic. I^2^ statistic below 40% and above 50% were classified as low and high heterogeneities, respectively [34]. A random-effects model was applied in the presence of high or low statistical heterogeneity for the between-intervention meta-analysis. Studies were categorized according to whether participants were matched for potential confounders (matched vs not matched), such as age, sex, and BMI, and pooled separately to examine the influence of confounder control on effect estimates. A chi-square test for subgroup differences (test for interaction) was used to examine whether effect estimates differed between matched and non-matched. Meta-regression analyses were employed to investigate sources of heterogeneity [34], including mean age (years), and BMI (kg·m^−2^). Sensitivity analyses were performed by sequentially excluding each predefined category individually (i.e., not simultaneously), including interventional studies, studies rated as low methodological quality, those that did not specify which first-degree relatives (parents and/or siblings) were considered, those with a mean participant age ≥ 40 years, and single-sex samples. Publication bias was evaluated for the primary outcomes using funnel plots. Funnel plot asymmetry was further examined using Begg’s and Egger’s tests [35] performed in JAMOVI software (version 1.6, Sydney, Australia). In the presence of significant funnel plot asymmetry, a trim-and-fill analysis was performed to estimate the number of potentially missing studies and to evaluate the potential impact of publication bias on the pooled effect estimates. The certainty of evidence for each outcome was assessed using the GRADE approach (Grading of Recommendations Assessment, Development and Evaluation), considering risk of bias, inconsistency, indirectness, imprecision, and publication bias. Observational studies started as low-certainty evidence and were downgraded when appropriate Meta-analyses were performed using the Review Manager software (RevMan 5.4, Copenhagen, Denmark). Effect estimates were calculated as mean differences based on the absolute values observed between groups (FHD^+^ and FHD^−^). Statistical significance was established at *p* ≤ 0.05.

## 3. Results

**Figure 1:**
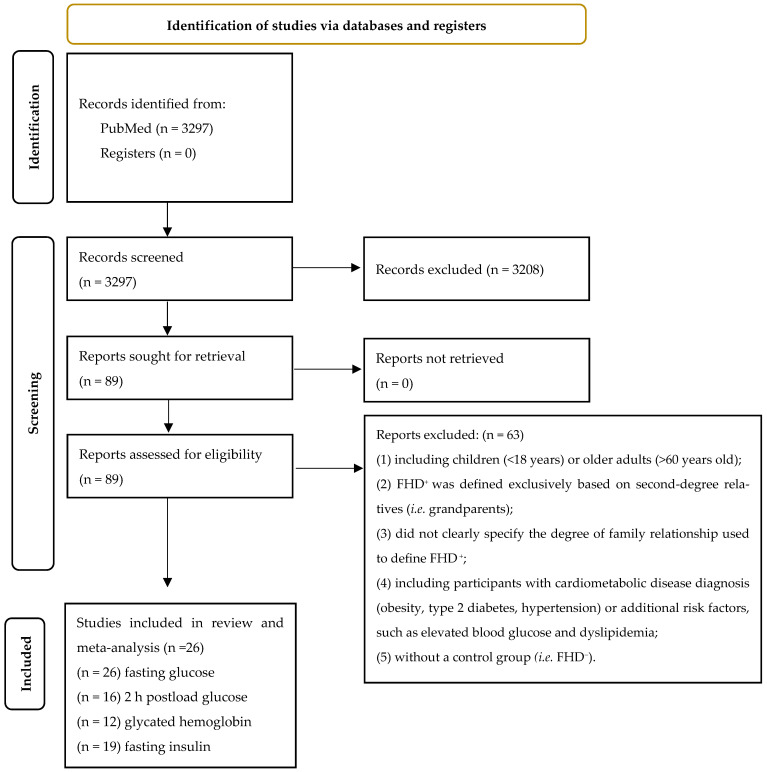
PRISMA flowdiagram.

The database search identified 3297 studies. After screening, 3208 were excluded because they did not satisfy the eligibility criteria. The full texts of the remaining 89 studies were subsequently assessed, of which 63 were excluded for failing to meet the inclusion criteria. Among the 26 studies included in the quantitative analysis, with 3122 participants, ~33 years old, the most (14 studies) included reported being matched for some confounder factors (i.e., age, sex, BMI) [6,9,12,13,14,15,17,20,21,22,23,24,28,30,36]. Of all the included studies, 26 assessed fasting glucose, 16 measured 2 h postload glucose, 12 examined A1c, 19 evaluated fasting insulin, 13 calculated HOMA–IR, and only one investigated HRV index [6]. The definition of FHD^+^ varied across studies. Fifteen studies defined FHD^+^ as having at least one affected parent, including two studies that required both parents to be affected. Ten studies did not specify which first-degree relatives (FDRs) were considered (i.e., parents and/or siblings), whereas one study defined FHD^+^ exclusively based on affected siblings (Table 1). Figure 1 shows a PRISMA flow diagram of selected studies, and Table 1 summarizes the main characteristics of all selected studies. Reporting of ethnicity and lifestyle characteristics was limited. Ethnicity was described in four studies [23,25,30,36]. Physical activity levels were reported in two studies [25,30], and smoking habits in three studies [23,24,36]. In contrast, some studies considered smoking [9,11,12,26,31] and alcohol consumption [12,23,31,36] as exclusion criteria rather than reporting these variables descriptively. Most studies [6,11,12,14,15,16,19,21,22,23,24,25,26,29,30,31,36] clearly reported that the use of medication was an exclusion criterion. The most included studies are cross-sectional (23); one is prospective [15], and two are interventional [24,30]. The most included studies (23) showed an average age below 40 years, and only four studies [6,10,13,15] showed an average age above 40 years. The most (21 studies) included both sexes [6,7,9,10,12,14,15,16,17,18,19,20,21,22,23,24,28,30,31,36], two included only women [25,29], and three included only men [11,13,26].

**Figure 2:**
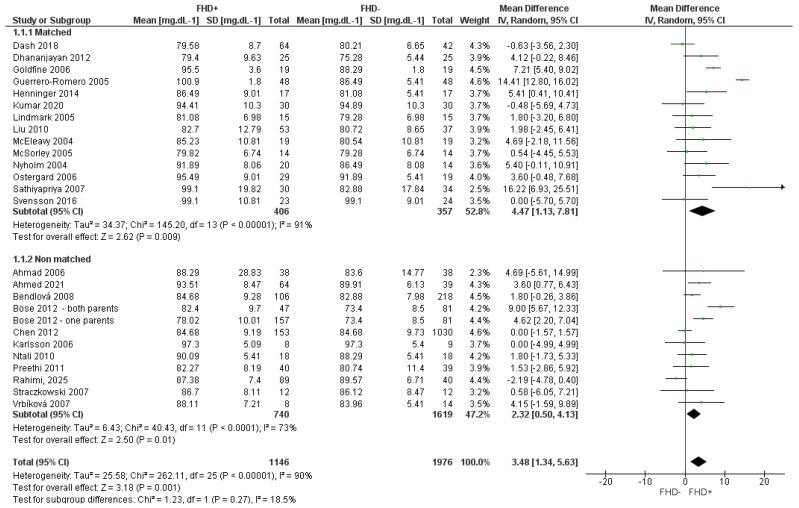
Fasting blood glucose in adults with a family history of diabetes (FHD^+^) and FHD^−^.

**Figure 3:**
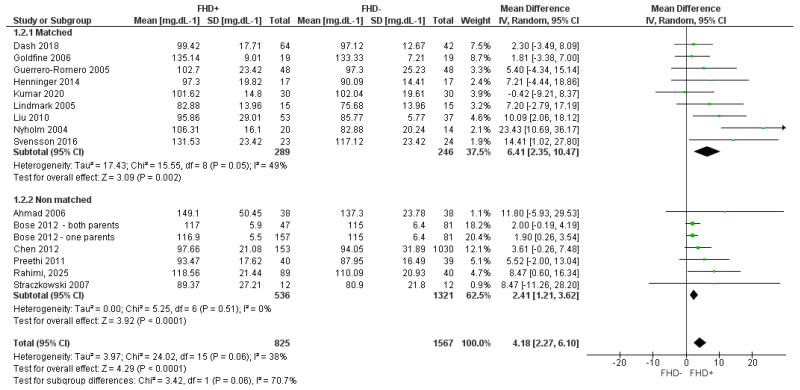
2 h postload glucose in adults with a family history of diabetes (FHD^+^) and FHD^−^.

**Figure 4:**
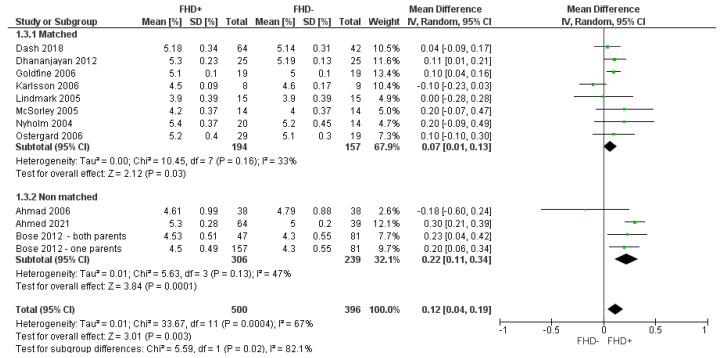
Glycated hemoglobin in adults with a family history of diabetes (FHD^+^) and FHD^−^.

**Figure 5:**
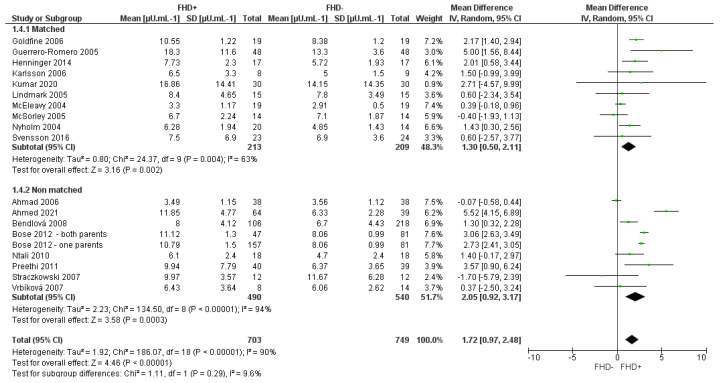
Fasting insulin in adults with a family history of diabetes (FHD^+^) and FHD^−^.

**Figure 6:**
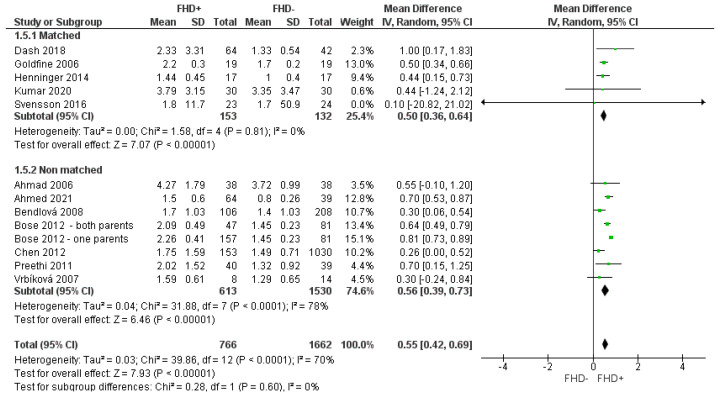
HOMA–IR in adults with a family history of diabetes (FHD^+^) and FHD^−^.

As shown in Figure 2, Figure 3 and Figure 4, the fasting glucose [3.48 (1.34–5.63) mg·dL^−1^; *p* < 0.001; n = 3122 individuals; I^2^ = 90%, *p* < 0.001], 2 h postload glucose [4.18 (2.27–6.10) mg·dL^−1^; *p* < 0.001; n = 2392 individuals; I^2^ = 38%, *p* = 0.06] and A1c [0.12 (0.04–0.19) %; *p* = 0.003; n =896 individuals; I^2^ = 67%, *p* < 0.001] were significantly higher in adults with an FHD^+^. The fasting insulin [1.72 (0.97–2.48) µU·mL^−1^; *p* < 0.001; n = 1452 individuals; I^2^ = 90%, *p* < 0.001] shown in Figure 5, and HOMA–IR [0.55 (0.42–0.69); *p* < 0.001; n = 2428 individuals; I^2^ = 70%, *p* < 0.001] shown in Figure 6 were also significantly higher in adults with an FHD^+^. The methodological quality of studies ranged from moderate (5) to high (9), with a median of seven points, and no study was considered unsatisfactory. The outcome domain showed consistently high quality across studies, whereas information regarding non-respondents was consistently lacking. In addition, the sample size justification and exposure assessment (FHD^+^ was predominantly based on self-report) were important limitations.

In addition, 26 and 18 studies reported BMI data and BP, respectively. BMI [0.93 (0.60–1.26) kg·m^−2^; *p* < 0.001; n = 3063 individuals; I^2^ = 35%, *p* = 0.04] was significantly higher in adults with FHD^+^ as shown in Figure S1. There were no differences to systolic BP [0.31 mmHg (−0.48–1.09); *p* = 0.45; n = 2546 individuals; I^2^ = 47%, *p* = 0.01] and diastolic BP [0.36 mmHg (−0.23–0.95); *p* = 0.23; n = 2480 individuals; I^2^ = 6%, *p* = 0.39] between groups, as shown in Figures S2 and S3.

Although pooled analyses were based on unadjusted group means, subgroup analyses were performed to examine whether methodological differences in confounder control influenced the results. Subgroup analyses showed that the overall effect direction remained consistent across studies that were matched groups for confounders. However, the magnitude of the association was attenuated for A1c in the subgroup of matched studies (test for subgroup differences, *p* < 0.05). In addition, heterogeneity was reduced in the matched subgroup for A1c, insulin, HOMA-IR, and BMI, suggesting that confounder control may partially explain between-study variability. Meta-regression analyses showed no significant associations between mean age or BMI and markers of glucose homeostasis in young adults, with β coefficients ranging from −0.314 to 0.435 across outcomes (Table 2).

Sensitivity analyses were conducted, excluding separately interventional studies, studies that did not specify the first-degree relatives (parents and/or siblings) considered in the definition of FHD^+^, and studies that classified FHD^+^ solely based on affected siblings, those with a mean participant age ≥ 40 years, and single-sex samples, and although the overall effect direction remained consistent (Table 3), the heterogeneity was reduced for 2 h postload glucose and A1c when considered only parents as first-degree relatives, suggesting that confounder may partially explain between-study variability. Sensitivity analyses based on methodological quality were not performed, as no included study was rated as unsatisfactory (≤4 points).

For most primary outcomes (see Supplementary Figures S4, S6–S8), neither the Begg’s rank correlation nor the Egger’s regression test indicated any funnel plot asymmetry (*p* > 0.05). However, for 2 h postload glucose, both the Begg’s rank correlation and Egger’s regression test indicated significant funnel plot asymmetry (*p* < 0.05) (Figure S5). The trim-and-fill procedure estimated that seven studies were potentially missing, suggesting the presence of publication bias. Rosenthal’s fail-safe N indicated that 213 additional null studies would be required to render the pooled effect non-significant. However, the adjusted pooled mean difference was attenuated from 4.18 to 2.61 mg·dL^−1^ (95% CI: 0.43 to 4.79; *p* = 0.019), although the association remained statistically significant (Table S2). The certainty of evidence was predominantly downgraded due to heterogeneity among studies, while evidence of publication bias was detected in only one outcome (Supplementary Table S3).

## 4. Discussion

In this meta-analysis, the FHD^+^ in healthy adults was associated with differences in glucose homeostasis compared to those without FHD^+^. Specifically, we found significantly higher levels of fasting glucose, 2 h postload glucose, A1c, fasting insulin, and HOMA–IR in FHD^+^ individuals.

Preventing new cases of T2DM could result in important public health improvements. Therefore, meta-analyses like this can orient researchers and health practitioners in developing strategies (i.e., diet and physical activity) to prevent or delay the onset of T2DM in susceptible individuals. Notably, family history should be interpreted as a complex exposure, encompassing not only genetic susceptibility but also shared environmental influences, including dietary patterns, lifestyle habits, socioeconomic factors, and household lifestyle patterns, all of which may contribute to the increased risk of T2DM. In this context, a positive parental history of diabetes has been associated with an increased risk of developing T2DM, regardless of age, sex, or adiposity level [37]. However, this risk appears even greater in lean individuals than in those who are overweight.

The global prevalence of fasting glucose and impaired glucose tolerance was estimated at 9.2% and 12%, respectively, of the adult population in 2024 [1]. These glycemic impairments substantially increased the likelihood of progressing to T2DM [38]. Over 10 years (30 to 40 years), a relative reduction in the initial glucose effectiveness in individuals with FHD^+^ contributes to the development of isolated impaired fasting glucose and, to a lesser extent, glucose intolerance and to future acute cell dysfunction [27]. Another study demonstrated that individuals between 28 and 45 years of age have a significant increase in predictors for T2DM in relation to the previous decade of life.

Ahmed et al. [11] reported insulin resistance with compensatory β–cell function (hyperinsulinemia) in FHD^+^ individuals, suggesting that insulin resistance precedes the development of pancreatic β–cell dysfunction in individuals at risk of T2DM. Similarly, Acevedo-Negrete et al. [39] reported that having at least one diabetic parent was associated with a 1.53-fold higher risk of developing hyperinsulinemia and a 1.47-fold higher risk of insulin resistance, even after adjusting for age, sex, BMI, fasting glucose, and triglycerides. Among individuals who developed DM, the levels of fasting and postload glucose and insulin secretion were higher, and insulin sensitivity was lower than that of the controls, even 13 years before the DM diagnosis. Yearly increases of 0.02–0.8 mmol·L^−1^·year^−1^ (0.4–14.4 mg·dL^−1^·year^−1^) in glucose were observed near diagnosis. [40]. A cohort study [41] showed that increased HOMA–IR in normoglycemic individuals is associated with increased incidence of pre-diabetes in both isolated impaired fasting glucose and isolated impaired glucose tolerance subtypes.

In addition, a study [42] describes the natural history of insulin secretion and insulin sensitivity in the development of pre-diabetes; the proportion of men and individuals with FHD^+^ was highest in the groups who later developed isolated impaired fasting glycemia and combined impaired fasting glycemia/impaired glucose tolerance. In addition, even modest hyperglycemia, still within the normal range, may contribute to impaired endothelial function and increased risk of early atherosclerotic changes. Goldfine et al. [9] found that minimal increases in fasting glucose significantly impacted endothelial function, even among individuals without diabetes. Likewise, Prado et al. [43] identified increased carotid intima-media thickness in FHD^+^ individuals, especially in the left common carotid artery, despite the absence of functional vascular change.

Various mechanisms have been proposed to explain elevated blood glucose levels in individuals with FHD^+^: for example, in insulin-resistant first-degree relatives of T2DM patients, insulin-stimulated phosphorylation of insulin receptor substrate-1 (IRS-1), phosphatidylinositol 3-kinase activity, and AKT phosphorylation are all impaired [44]. In addition, another study [13] observed that insulin-mediated glucose uptake in skeletal muscle from glucose-tolerant first-degree relatives of T2DM patients is impaired, despite unchanged phosphorylation of Akt and AS160. The uncoupling of insulin action on AS160 and glucose transport may constitute an early defect in the pathogenesis of T2DM. Additionally, sympathetic activation has been considered a link between insulin resistance, hyperinsulinemia, and T2DM risk [45,46]. Frontoni et al. [45] suggested that insulin resistance and compensatory hyperinsulinemia, and enhanced sympathetic activity are the primary events leading to the development of diabetes in FHD^+^. Only one eligible study evaluated HRV [6]. This study reported lower time-domain indices (RMSSD and pNN50) in individuals with FHD^+^, whereas no differences were observed in frequency-domain indices (LF, HF, and LF/HF). Consequently, a meta-analysis could not be performed.

Risk factors such as obesity can exacerbate an underlying genetic susceptibility to T2DM. Although obesity was an exclusion criterion in our study, individuals with FHD^+^ still exhibited higher BMI values. Related to this, Kumar et al. [28] reported impaired postprandial metabolism and insulin sensitivity in overweight normoglycemic young adults without FHD^+^. In addition, the higher BMI observed in FHD^+^ individuals may contribute to alterations in inflammatory levels. FHD^+^ individuals have increased obesity, insulin resistance, and dyslipidemia [47]. Chronic inflammation plays a central role in insulin resistance, T2DM, and endothelial dysfunction [20]. Tesauro et al. [48] found elevated levels of inflammatory markers, such as CRP, TNF-α, and IL1-β, in FHD^+^ individuals. Moreover, some studies have suggested an imbalance in the oxidant/antioxidant ratio (i.e., erythrocyte glutathione peroxidase, lipid peroxidation, and protein carbonyl) among first-degree relatives of T2DM patients [14]. Henninger et al. [17] demonstrated that even in non-obese, glucose-tolerant individuals with FHD^+^, early signs of remodeling in the abdominal subcutaneous adipose tissue (adipocyte hypertrophy and inflammation) were already present, resembling an “obese phenotype.”

From a clinical perspective, it is worth noting that, although the pooled differences were statistically significant, their clinical relevance requires careful consideration because the observed differences were modest in absolute terms and, in most cases, remained within clinically normal ranges. While these small differences are unlikely to have immediate clinical implications at the individual level, subtle shifts in glycemic markers at the population level may still be relevant from a public health perspective, particularly if they precede the development of more pronounced metabolic abnormalities. Prospective longitudinal studies are needed to determine whether these modest differences translate into an increased risk of prediabetes, type 2 diabetes, or cardiovascular events over time.

An important limitation of this review is that the literature search was restricted to articles in English and those found in the PubMed/MEDLINE database, which may have excluded some potentially relevant data published in other languages and databases. Consequently, selection and language bias cannot be excluded, and the comprehensiveness and generalizability of the findings may have been affected. In addition, our findings are limited to adults without comorbidities, and the observed outcomes were based mainly on cross-sectional studies (~88% of studies), which did not allow the establishment of a causal relationship between FHD^+^ and glucose homeostasis. However, none of these studies were considered of unsatisfactory quality by NOS. Although study design, methodological quality, specificity of first-degree relatives, age, and sex may act as potential covariates, sensitivity analyses yielded similar results after excluding interventional studies, those that did not specify which first-degree relatives (parents and/or siblings) were considered, studies with a mean participant age ≥ 40 years, and single-sex samples. Sensitivity analyses based on methodological quality were not performed because no included study was rated as unsatisfactory. Subgroup analyses according to these covariates were not conducted due to the small number of studies within each category, which limited statistical power. Meta-regression analyses showed no significant associations between mean age or BMI and markers of glucose homeostasis. The various sensitivity analyses performed did not reduce heterogeneity for fasting glucose and fasting insulin, indicating that the sources of heterogeneity for these outcomes remain largely unexplained. Another limitation is that the pooled analyses were based on unadjusted group means, as adjusted estimates were not consistently available across the included studies. In addition, ethnicity, physical activity, and smoking status were reported in only a few studies. The lack of data limits the ability to exclude residual confounding, even in matched studies, and may have influenced the observed associations. Other factors, including geographical region, study period, fasting state verification protocols, and insulin assay methods, may have contributed to the observed between-study variability but could not be systematically explored because these data were inconsistently reported across studies. These limitations should be considered when interpreting the pooled estimates. Regarding studies evaluating 2 h postload glucose, evidence of funnel plot asymmetry was found, potentially suggesting the occurrence of publication bias and small-study effects. Although the trim-and-fill analysis attenuated the pooled effect estimate, the association remained statistically significant after inputting the potentially missing studies. In this context, results for this outcome should be interpreted with caution. Although the certainty of evidence was rated as very low according to GRADE criteria, this is common in meta-analyses of observational studies, particularly due to methodological heterogeneity and the inherent limitations of non-randomized designs. In a recent cohort study of patients with diabetes, it was found that 78% and 53% of patients with early onset (age 20–45 years) and with normal onset T2DM (age > 50 years), respectively, were men [49]. Further research is required to confirm our findings and investigate the potential role of FHD^+^ on glucose levels, insulin, insulin resistance, cardiac autonomic modulation, and sex-related differences. Identifying individuals who already exhibit subtle alterations in glucose homeostasis, before the stage currently defined as prediabetes according to the American Diabetes Association [38], would lead to better preventive outcomes for β-cell function.

## 5. Conclusions

In summary, the available evidence, derived predominantly from observational studies, suggests that FHD^+^ may be associated with markers of impaired glucose homeostasis in healthy adults. These findings are consistent with the hypothesis that early metabolic alterations may be present in at-risk individuals, potentially years before conventional diagnostic criteria are met. However, these results should be interpreted with caution and confirmed in higher-quality prospective studies.

## Figures and Tables

**Table 1:** Characteristics of included studies separated by matched studies (for age, sex, BMI, or WHR) and non-matched studies.

Author (Year)	Country	Study Design	Family History	Matched Group	N	Sex (M/W)	Age (Range or Mean ± SD–FHD^−^/FHD^+^)	BMI (FHD^−^/FHD^+^)	Medication Use
Matched studies									
Dash 2018 [20]	India	cross-sectional	≥1 parent	age, sex, BMI	108	both (81/25)	20–40	23.28 ± 3.48/24.41 ± 4.09	NR
Dhananjayan 2013 [21]	Índia	cross-sectional	≥1 parent	age, sex	50	both	26.92 ± 2.63/26.16 ± 3.18	23.66 ± 4.33/22.19 ± 1.8	excluded
Goldfine 2006 [9]	United States	cross-sectional	both parents	age, sex, BMI	38	both (18/20)	36.5 ± 2/38 ± 2	21.6 ± 1.2/27 ± 1	NR
Guerrero-Romero 2005 [12]	México	cross-sectional	≥1 parent	age, sex, WHR	96	both (36/60)	19.8 ± 2.6/19.4 ± 3.6	21 ± 1.9/21.7 ± 2.1	excluded
Henninger 2014 [17]	Sweden	cross-sectional	≥1 FDR	age, sex, BMI	34	both (12/22)	34 ± 8/38 ± 9	24.1 ± 2.37/24.7 ± 2.31	NR
Kumar 2020 [28]	Índia	cross-sectional	≥1 FDR	age, sex	60	both (30/30)	18–40	normal weight	NR
Lindmark, 2005 [22]	Sweden	cross-sectional	≥1 FDR	age, sex, BMI	30	both (16/14)	32.7 ± 1.9/32.5 ± 1.9	23.6 ± 0.7/24.2 ± 0.9	excluded
Liu 2010 [15]	China	prospective	≥1 FDR	age, BMI, WHR	90	both (35/55)	49.19 ± 8.76/48.55 ± 7.30	24.49 ± 3.47/24.72 ± 3.15	excluded
McEleavy 2004 [23]	United Kingdon	cross-sectional	≥1 parent	age, sex, BMI	38	both (16/22)	32.8 ± 9.5/33.1 ± 9.6	24.3 ± 3.4/24.8 ± 4.9	excluded
McSorley 2005 [24]	Ireland	interventional	≥1 parent	similar characteristics	28	both (24/4)	18–38	24.9 ± 2.99/24.5 ± 3.74	excluded
Nyholm 2004 [36]	Denmark	cross-sectional	≥1 FDR	age, sex, BMI	34	both (17/17)	34.6 ± 7.86/34.8 ± 9.84	24.8 ± 2.62/25.2 ± 2.24	excluded
Ostergard 2006 [30]	Denmark	interventional	≥1 parent	age, sex, BMI	48	both (33/15)	31 ± 5/33 ± 5	25.8 ± 3.0/26.3 ± 1.6	excluded
Sathiyapriya 2007 [14]	India	cross-sectional	≥1 FDR	age	64	both (39/25)	33.02 ± 8.4/36.1 ± 8.8	23.3 ± 2.9/24.2 ± 3.4	excluded
Svensson 2016 [6]	Sweden	cross-sectional	≥1 FDR	age, sex, BMI	47	both (25/22)	47.2 ± 11.7/46.8 ± 12.0	25.0 ± 3.1/25.1 ± 3.8	excluded
Non-matched studies									
Ahmad 2006 [19]	India	cross-sectional	≥1 parent	NR	76	both (59/17)	28.30 ± 3.66/30.84 ± 4.64	21.06 ± 5.29/25.84 ± 2.63	excluded
Ahmed 2021 [11]	Republic of Yemen	cross-sectional	≥1 FDR	NR	103	men	31.4 ± 4.61/32.3 ± 5.16	21.2 ± 2.52/22.9 ± 2.11	excluded
Bendlová 2008 [16]	Czech Republic	cross-sectional	≥1 parent	NR	99	men	29.4 ± 8.12/39.4 ± 8.62	24.20 ± 3.25/25.8 ± 3.45	excluded
Bendlová 2008 [16]	Czech Republic	cross-sectional	≥1 parent	NR	225	women	29.5 ± 11.1/36.9 ± 12.82	23.1 ± 3.7/24.3 ± 3.42	excluded
Bose 2012—both parents [7]	India	cross-sectional	both parents	NR	128	both	18–22	21.8 ± 3.3/23.6 ± 3.5	NR
Bose 2012—one parent [7]	India	cross-sectional	1 parent	NR	238	both	18–22	21.8 ± 3.3/22.4 ± 4.1	NR
Chen 2012 [10]	China	cross-sectional	≥1 parent	NR	1183	both (406/777)	40.57 ± 12.56/39.59 ± 8.68	22.58 ± 3.16/23.19 ± 3.1	NR
Karlsson, 2006 [13]	Finland	cross-sectional	≥1 FDR	NR	17	men	40 ± 2/41 ± 3	23.4 ± 0.7/25.1 ± 0.8	NR
Ntali, 2010 [25]	UK	cross-sectional	≥1 parent	NR	36	women	30.1 ± 6.8/30.6 ± 6.5	22.2 ± 1.8/22.1 ± 2.5	excluded
Preethi 2010 [18]	India	cross-sectional	siblings	NR	79	both (33/47)	18.90 ± 1.58/19.10 ± 1.83	21.31 ± 3.04/23.34 ± 4.75	NR
Rahimi 2025 [31]	Iran	cross-sectional	≥1 parent	NR	129	both (49/80)	29.49 ± 5.84/30.15 ± 7.61	23.80 ± 2.96/25.34 ± 4.05	excluded
Straczkowski 2007 [26]	Poland	cross-sectional	≥1 parent	NR	24	men	28.17 ± 7.07/27.75 ± 6.06	22.74 ± 1.51/22.33 ± 2.34	excluded
Vrbíková 2007 [29]	Czech Republic	cross-sectional	≥1 FDR	NR	22	women	24.2 ± 4.8/27.5 ± 3.2	22.52 ± 2.80/23.54 ± 6.04	excluded

BMI: body mass index; Excluded: it was included in the study exclusion criteria; FDR: at least one first-degree relative (parent and/or sibling), without specification of which relative was affected; N: number of participants; NR: not reported; WHR: waist-to-hip ratio.

**Table 2:** Meta-regression results.

Outcome/Moderator	Number of Study Estimates	β	95% Confidence Interval	*p*	R2
Fasting Glucose (mg·dL^−1^)					
Age (years)	24	−0.314	−1.216 to 0.589	0.496	0.00
Body mass index (kg·m^−2^)	26	−0.113	−2.557 to 2.330	0.927	0.00
2 h Postload Glucose (mg·dL^−1^)					
Age (years)	13	−0.057	−2.384 to 2.269	0.961	0.00
Body mass index (kg·m^−2^)	15	0.435	−2.462 to 3.332	0.768	0.00
A1c (%)					
Age (years)	10	0.004	−0.091 to 0.010	0.931	0.00
Body mass index (kg·m^−2^)	12	0.043	−0.047 to 0.133	0.352	0.00
Insulin (µU·dL^−1^)					
Age (years)	15	0.193	−0.530 to 0.916	0.600	0.00
Body mass index (kg·m^−2^)	17	−0.093	−0.712 to 0.527	0.769	0.00
HOMA					
Age (years)	9	0.003	−0.089 to 0.095	0.949	0.00
Body mass index (kg·m^−2^)	9	−0.039	−0.195 to 0.117	0.624	0.00

**Table 3:** Sensitivity analyses, including only cross-sectional studies, those that considered at least one parent as a first-degree relative, those with a mean participant age < 40 years, and both-sex samples.

Variable	Studies	N	– (%)	Mean Difference (95% IC)	*p*
Fasting glucose (mg·dL^−1^)					
Design studies (cross-sectional)	23	2956	91	3.67 [1.33, 6.02]	0.002
≥1 parent	16	2629	94	4.15 [1.16, 7,13]	0.006
Age (<40 years)	22	1785	90	4.04 [1.66, 6.42]	<0.001
Sex (both)	21	2920	92	3.82 [1.30, 6.34]	0.003
2 h postload glucose (mg·dL^−1^)					
Design studies (cross-sectional)	15	2302	33	3.75 [1.91, 5.60]	<0.001
≥1 parent	9	2018	0	2.35 [1.19, 3.50]	<0.001
Age (<40 years)	13	1072	32	3.54 [1.55, 5.52]	<0.001
Sex (both)	15	2368	41	4.20 [2.24, 6.16]	<0.001
A1c (%)					
Design studies (cross-sectional)	10	820	73	0.11 [0.03, 0.20]	0.01
≥1 parent	8	712	0	0.11 [0.07, 0.15]	<0.001
Age (<40 years)	11	879	52	0.14 [0.08, 0.21]	<0.001
Sex (both)	10	776	0	0.11 [0.07, 0.15]	<0.001
Fasting insulin (µU·mL^−1^)					
Design studies (cross-sectional)	18	1424	90	1.86 [1.08, 2.63]	<0.001
≥1 parent	10	1026	94	1.43 [0.47, 2.39]	0.004
Age (<40 years)	17	1388	91	1.77 [0.98, 2.57]	<0.001
Sex (both)	14	1250	92	1.62 [0.79, 2.45]	<0.001
HOMA–IR					
Design studies (cross-sectional)	13	2428	70	0.55 [0.42,0.69]	<0.001
≥1 parent	7	2083	83	0.55 [0.35, 0.74]	<0.001
Age (<40 years)	11	1198	66	0.59 [0.46, 0.72]	<0.001
Sex (both)	12	2303	74	0.54 [0.38, 0.70]	<0.001

N: number of participants.

## Data Availability

No new data were created or analyzed in this study.

## Supplementary Materials

The following supporting information can be downloaded at https://www.mdpi.com/article/10.3390/medsci14030349/s1. Table S1. Eligibility criteria for the meta-analysis; Figure S1. Body mass index (BMI) in adults with a family history of diabetes (FHD^+^) and FHD^−^; Figure S2. Systolic blood pressure (BP) in adults with a family history of diabetes (FHD^+^) and FHD^−^; Figure S3. Diastolic blood pressure (BP) in adults with a family history of diabetes (FHD^+^) and FHD^−^; Figure S4. Funnel plot bias assessment of overall studies reporting fasting glucose as the outcome. Begg’s (*p* = 0.382) and Egger’s (*p* = 0.812); Figure S5. Funnel plot bias assessment of overall studies reporting 2-h postload glucose as the outcome. Begg’s (*p* = 0.006) and Egger’s (*p* < 0.001); Table S2. Publication bias assessment for the 2-h postload glucose outcome; Figure S6. Funnel plot bias assessment of overall studies reporting A1c as the outcome. Begg’s (*p* = 0.638) and Egger’s (*p* = 0.539); Figure S7. Funnel plot bias assessment of over-all studies reporting fasting insulin as the outcome. Begg’s (*p* = 0.945) and Egger’s (*p* = 0.839); Figure S8. Funnel plot bias assessment of overall studies reporting HOMA as the outcome. Begg’s (*p* = 0.590) and Egger’s (*p* = 0.656); Table S3. Certainty of evidence according to the GRADE approach. File S1. PRISMA 2020 Checklist.

## Abbreviations

The following abbreviations are used in this manuscript:

A1c	Glycated hemoglobin
BMI	Body mass index
BP	Blood pressure
FHD^+^	Family history of diabetes mellitus
GRADE	Grading of recommendations, assessment, development, and evaluation
HOMA–IR	Homeostatic model assessment for insulin resistance
HRV	Heart rate variability
NOS	Newcastle–Ottawa Scale
PICOS	Population, Intervention/Exposure, Comparison, Outcome, and Study
PROSPERO	Prospective Register of Systematic Reviews
SEM	Standard error of the mean
T2DM	Type 2 diabetes mellitus
