# Supplementary material for: Early Alterations in Glucose Homeostasis Associated with a Family History of Diabetes Mellitus: A Systematic Review and Meta-Analysis
Source: Med Sci (Basel). 2026 Jun 26;14(3):349. doi: 10.3390/medsci14030349 (PMC13414045; doi:10.3390/medsci14030349)
Supplement: Supplementary file 1 [file medsci-14-00349-s001.zip › medsci-4360308-supplementary.pdf]

Table S1. Eligibility criteria for the meta-analysis

PICOS Element	Description
**Population (P)**	Adults aged 18–60 years, both sexes, without diagnosed cardiometabolic diseases (obesity, type 2 diabetes, hypertension) and without additional cardiometabolic risk factors (i.e. dyslipidemia).
**Intervention / Exposure (I)**	Family history of diabetes (FHD^+), defined as having at least one first-degree relatives (parents and/or siblings) with diabetes mellitus.
**Comparator (C)**	Individuals without family history of diabetes mellitus ($FHD^–$).
**Primary Outcomes (O)**	Fasting glucose, 2-hour postload glucose, glycated hemoglobin (A1c), fasting insulin, HOMA-IR, and heart rate variability (HRV) indices.
**Secondary Outcomes (O)**	Body mass index (BMI) and systolic and diastolic blood pressure (BP).
**Study Design (S)**	Observational studies (cross-sectional and prospective) and interventional studies (baseline data only).
**Language**	Full-text articles published in English

Study or Subgroup	FHD+ Mean [Kg.m-2]	FHD+ SD [Kg.m-2]	FHD+ Total	FHD- Mean [Kg.m-2]	FHD- SD [Kg.m-2]	FHD- Total	Weight	Mean Difference IV, Random, 95% CI
**2.1.1 Matched**								
Dash 2018	24.41	4.09	64	23.28	3.48	42	3.6%	1.13 [-0.32, 2.58]
Dhananjayan 2012	23.66	4.33	25	22.19	1.8	25	2.5%	1.47 [-0.37, 3.31]
Goldfine 2006	27	1	19	26.7	1.2	19	8.0%	0.30 [-0.40, 1.00]
Guerrero-Romero 2005	21.7	2.1	48	21	1.9	48	7.2%	0.70 [-0.10, 1.50]
Henninger 2014	24.7	2.31	17	24.1	2.37	17	3.2%	0.60 [-0.97, 2.17]
Karlsson 2006	25.1	2.26	8	23.4	2.1	9	2.1%	1.70 [-0.38, 3.78]
Lindmark 2005	24.2	3.49	15	23.6	2.71	15	1.8%	0.60 [-1.64, 2.84]
Liu 2010	24.72	3.15	53	24.49	3.47	37	3.8%	0.23 [-1.17, 1.63]
McEleavy 2004	24.8	4.9	19	24.3	3.4	19	1.3%	0.50 [-2.18, 3.18]
McSorley 2005	24.5	3.74	14	24.9	2.99	14	1.5%	-0.40 [-2.91, 2.11]
Nyholm 2004	25.2	2.24	20	24.8	2.62	14	2.9%	0.40 [-1.29, 2.09]
Ostergard 2006	26.3	1.6	29	25.8	3	19	3.6%	0.50 [-0.97, 1.97]
Sathiyapriya 2007	24.2	3.4	30	23.3	2.9	34	3.3%	0.90 [-0.66, 2.46]
Svensson 2016	25.1	3.8	23	25	3.1	24	2.2%	0.10 [-1.89, 2.09]
**Subtotal (95% CI)**			**384**			**336**	**47.0%**	**0.58 [0.21, 0.94]**
Heterogeneity: Tau² = 0.00; Chi² = 4.53, df = 13 (P = 0.98); I² = 0%								
Test for overall effect: Z = 3.09 (P = 0.002)								
**2.1.2 Non matched**								
Ahmad 2006	25.84	2.63	38	21.06	5.29	38	2.4%	4.78 [2.90, 6.66]
Ahmed 2021	22.9	2.11	64	21.2	2.52	39	6.1%	1.70 [0.76, 2.64]
Bendlová 2008 men	25.8	3.45	33	24.2	3.25	66	3.8%	1.60 [0.19, 3.01]
Bendlová 2008 women	24.3	3.42	73	23.1	3.7	152	5.9%	1.20 [0.22, 2.18]
Bose 2012 - both parents	23.6	3.5	47	21.8	3.3	81	4.5%	1.80 [0.57, 3.03]
Bose 2012 - one parents	22.4	4.1	157	21.8	3.3	81	6.0%	0.60 [-0.36, 1.56]
Chen 2012	23.19	3.1	153	22.58	3.16	1030	9.5%	0.61 [0.08, 1.14]
Ntali 2010	22.1	2.5	18	22.2	1.8	18	3.7%	-0.10 [-1.52, 1.32]
Preethi 2011	23.34	4.75	47	21.31	3.04	33	2.8%	2.03 [0.32, 3.74]
Rahimi, 2025	25.35	4.05	89	23.8	2.96	40	4.5%	1.55 [0.31, 2.79]
Straczkowski 2007	22.33	2.34	12	22.74	1.51	12	3.2%	-0.41 [-1.99, 1.17]
Vrbíková 2007	23.54	6.04	8	22.52	2.8	14	0.5%	1.02 [-3.41, 5.45]
**Subtotal (95% CI)**			**739**			**1604**	**53.0%**	**1.27 [0.69, 1.86]**
Heterogeneity: Tau² = 0.59; Chi² = 30.25, df = 11 (P = 0.001); I² = 64%								
Test for overall effect: Z = 4.27 (P < 0.0001)								
**Total (95% CI)**			**1123**			**1940**	**100.0%**	**0.93 [0.60, 1.26]**
Heterogeneity: Tau² = 0.22; Chi² = 38.71, df = 25 (P = 0.04); I² = 35%								
Test for overall effect: Z = 5.58 (P < 0.00001)								
Test for subgroup differences: Chi² = 3.91, df = 1 (P = 0.05), I² = 74.4%								

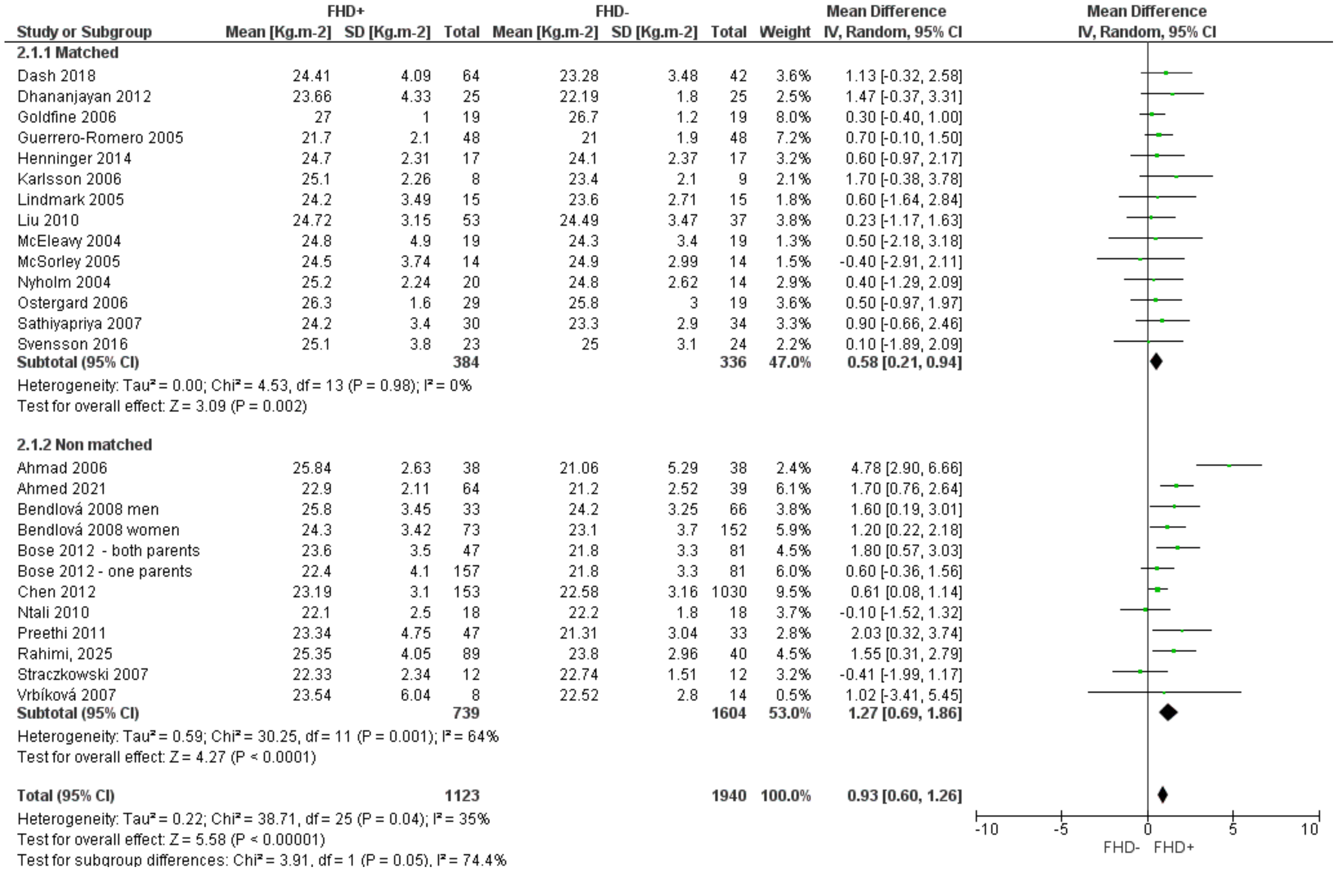

Figure S1. Body mass index (BMI) in adults with a family history of diabetes (FHD^+) and FHD^-.

Study or Subgroup	FHD+ Mean [mmHg]	FHD+ SD [mmHg]	FHD+ Total	FHD- Mean [mmHg]	FHD- SD [mmHg]	FHD- Total	Weight	Mean Difference IV, Fixed, 95% CI
**3.1.1 Matched**								
Dash 2018	119	9	64	118	8	42	5.7%	1.00 [-2.27, 4.27]
Dhananjayan 2012	117	7	25	119	2	25	7.6%	-2.00 [-4.85, 0.85]
Goldfine 2006	119	4	19	120	4	19	9.5%	-1.00 [-3.54, 1.54]
Guerrero-Romero 2005	110	11	48	106	17	48	1.9%	4.00 [-1.73, 9.73]
Karlsson 2006	134	8	8	125	6	9	1.3%	9.00 [2.21, 15.79]
Lindmark 2005	128	15	15	118	12	15	0.7%	10.00 [0.28, 19.72]
Liu 2010	122	16	53	123	14	37	1.6%	-1.00 [-7.24, 5.24]
McEleavy 2004	120	9	19	119	14	19	1.1%	1.00 [-6.48, 8.48]
McSorley 2005	118	11	14	121	7	14	1.3%	-3.00 [-9.83, 3.83]
Ostergard 2006	130	8	29	129	7	19	3.3%	1.00 [-3.29, 5.29]
Svensson 2016	118	18	23	113	11	24	0.8%	5.00 [-3.57, 13.57]
**Subtotal (95% CI)**			**317**			**271**	**34.9%**	**0.29 [-1.03, 1.62]**
Heterogeneity: Chi² = 17.76, df = 10 (P = 0.06); I² = 44%								
Test for overall effect: Z = 0.43 (P = 0.66)								
**3.1.2 Non matched**								
Ahmad 2006	124	10	38	118	9	38	3.4%	6.00 [1.72, 10.28]
Ahmed 2021	117	11	64	112	11	39	3.2%	5.00 [0.62, 9.38]
Bose 2012 - both parents	110	5	157	110	6	81	26.5%	0.00 [-1.52, 1.52]
Bose 2012 - one parents	110	7	47	110	6	81	10.8%	0.00 [-2.39, 2.39]
Chen 2012	114	15	153	115	30	1030	6.8%	-1.00 [-4.00, 2.00]
Preethi 2011	119	6	40	120	6	39	8.8%	-1.00 [-3.65, 1.65]
Rahimi, 2025	107	10	89	108	9	40	5.1%	-1.00 [-4.48, 2.48]
Vrbíková 2007	121	14	8	112	9	14	0.5%	9.00 [-1.79, 19.79]
**Subtotal (95% CI)**			**596**			**1362**	**65.1%**	**0.31 [-0.66, 1.28]**
Heterogeneity: Chi² = 16.14, df = 7 (P = 0.02); I² = 57%								
Test for overall effect: Z = 0.63 (P = 0.53)								
**Total (95% CI)**			**913**			**1633**	**100.0%**	**0.31 [-0.48, 1.09]**
Heterogeneity: Chi² = 33.90, df = 18 (P = 0.01); I² = 47%								
Test for overall effect: Z = 0.76 (P = 0.45)								
Test for subgroup differences: Chi² = 0.00, df = 1 (P = 0.98), I² = 0%								

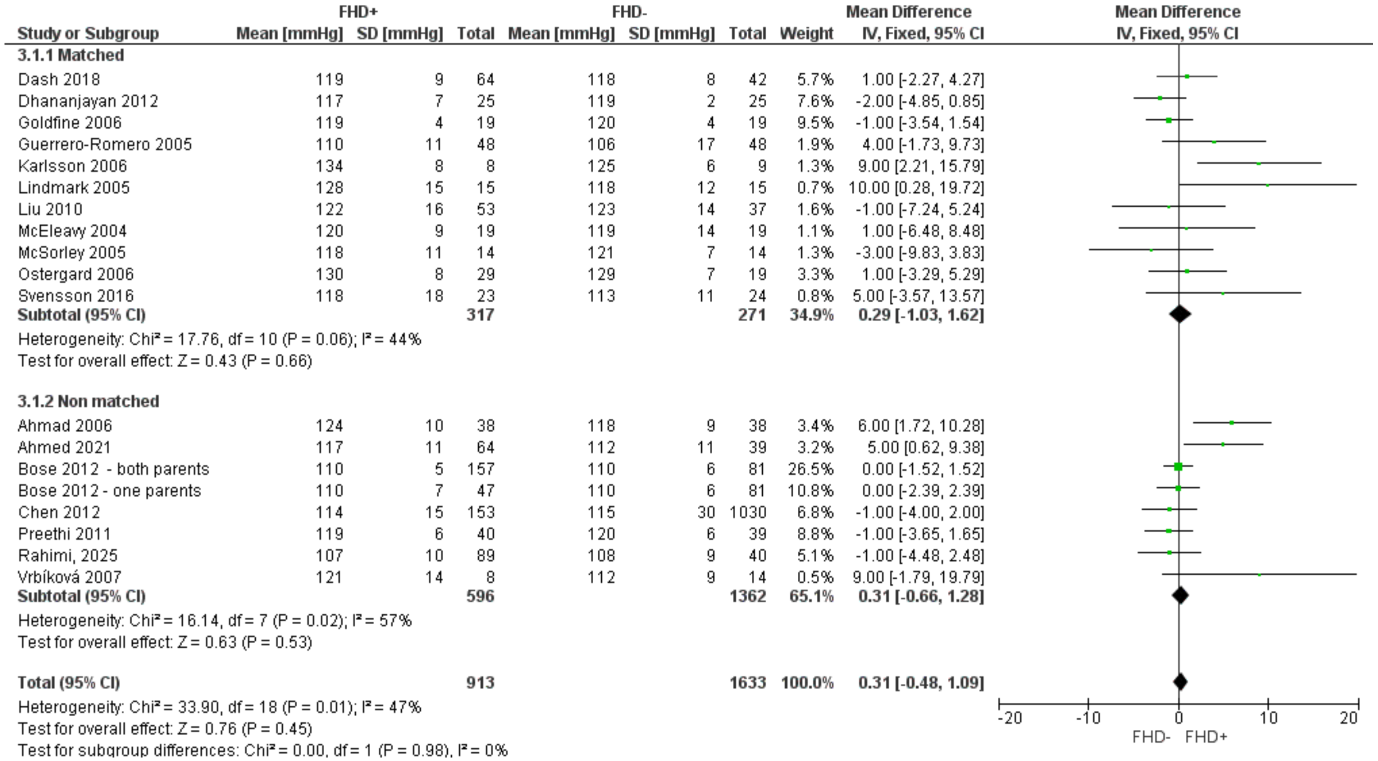

Figure S2. Systolic blood pressure (BP) in adults with a family history of diabetes (FHD^+) and FHD^-.

	FHD+			FHD-				Mean Difference
Study or Subgroup	Mean [mmHg]	SD [mmHg]	Total	Mean [mmHg]	SD [mmHg]	Total	Weight	IV, Random, 95% CI
**3.2.1 Matched**								
Dash 2018	76	6	64	75	7	42	5.0%	1.00 [-1.58, 3.58]
Dhananjayan 2012	81	8	25	79	2	25	3.2%	2.00 [-1.23, 5.23]
Goldfine 2006	70	2	19	70	2	19	17.7%	0.00 [-1.27, 1.27]
Guerrero-Romero 2005	72	10	48	72	12	48	1.8%	0.00 [-4.42, 4.42]
Karlsson 2006	82	8	8	78	6	9	0.7%	4.00 [-2.79, 10.79]
Lindmark 2005	76	8	15	76	8	15	1.1%	0.00 [-5.73, 5.73]
Liu 2010	78	11	53	77	8	37	2.2%	1.00 [-2.93, 4.93]
Ostergard 2006	78	4	29	77	3	19	8.1%	1.00 [-0.98, 2.98]
Svensson 2016	77	12	23	79	13	24	0.7%	-2.00 [-9.15, 5.15]
**Subtotal (95% CI)**			**284**			**238**	**40.4%**	**0.54 [-0.33, 1.42]**
Heterogeneity: Tau² = 0.00; Chi² = 3.43, df = 8 (P = 0.90); I² = 0%								
Test for overall effect: Z = 1.21 (P = 0.22)								
**3.2.2 Non matched**								
Ahmad 2006	76	8	38	75	7	38	3.0%	1.00 [-2.38, 4.38]
Ahmed 2021	77	8	64	73	8	39	3.3%	4.00 [0.81, 7.19]
Bose 2012 - both parents	76	4	47	75	4	81	14.4%	1.00 [-0.44, 2.44]
Bose 2012 - one parents	74	4	157	75	4	81	23.2%	-1.00 [-2.07, 0.07]
Chen 2012	75	10	153	75	30	1030	5.6%	0.00 [-2.42, 2.42]
Preethi 2011	74	5	40	74	5	39	6.7%	0.00 [-2.21, 2.21]
Rahimi, 2025	69	10	89	68	9	40	2.8%	1.00 [-2.48, 4.48]
Vrbíková 2007	75	8	8	71	8	14	0.7%	4.00 [-2.95, 10.95]
**Subtotal (95% CI)**			**596**			**1362**	**59.6%**	**0.58 [-0.55, 1.71]**
Heterogeneity: Tau² = 1.06; Chi² = 13.05, df = 7 (P = 0.07); I² = 46%								
Test for overall effect: Z = 1.01 (P = 0.31)								
**Total (95% CI)**			**880**			**1600**	**100.0%**	**0.36 [-0.23, 0.95]**
Heterogeneity: Tau² = 0.09; Chi² = 17.00, df = 16 (P = 0.39); I² = 6%								
Test for overall effect: Z = 1.21 (P = 0.23)								
Test for subgroup differences: Chi² = 0.00, df = 1 (P = 0.96), I² = 0%								

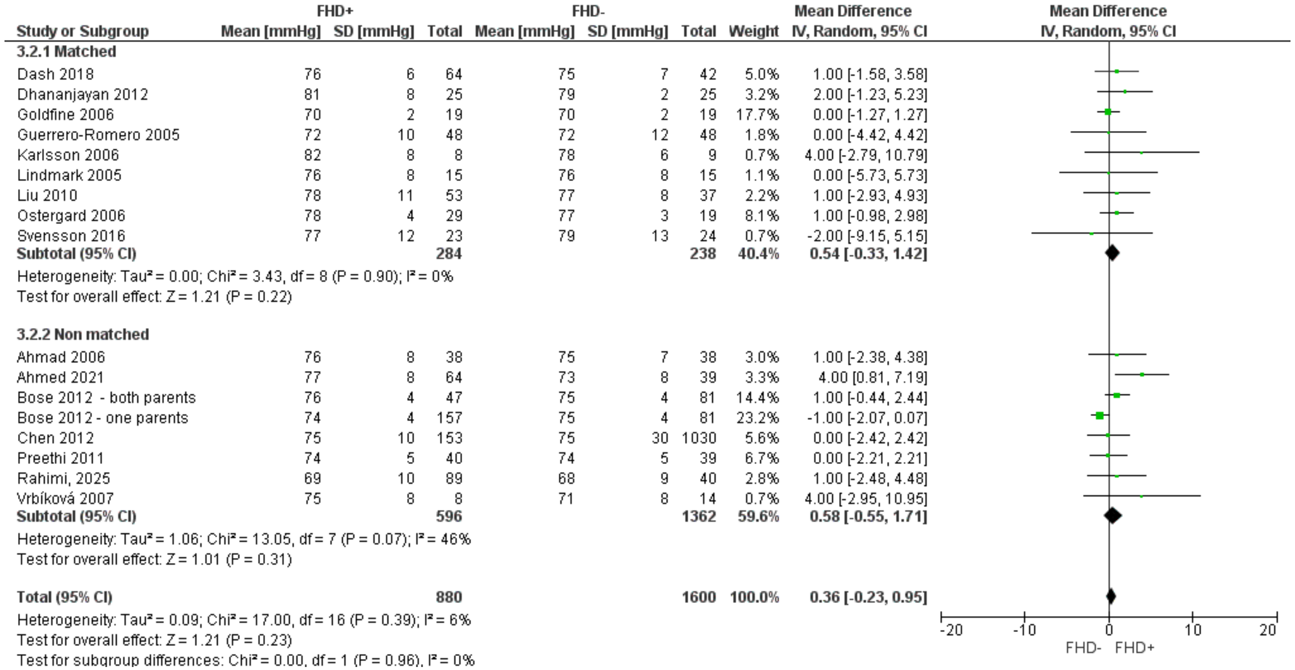

Figure S3. Diastolic blood pressure (BP) in adults with a family history of diabetes (FHD^+) and FHD^-

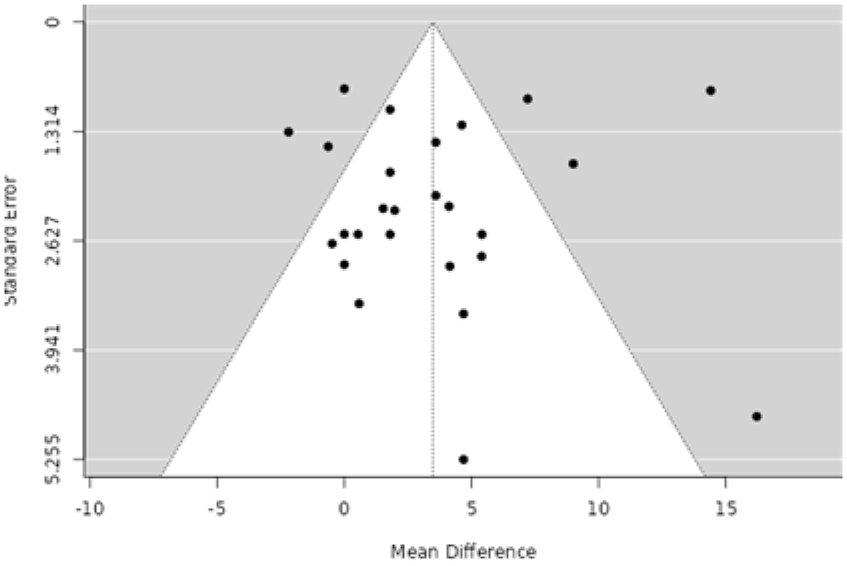

Figure S4. Funnel plot bias assessment of overall studies reporting fasting glucose as the outcome. Begg's (p = 0.382) and Egger's (p = 0.812).

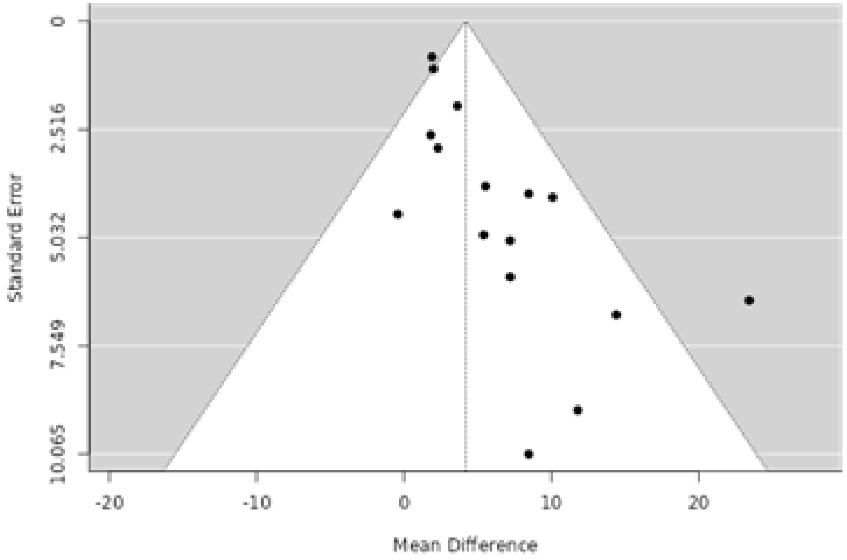

Figure S5. Funnel plot bias assessment of overall studies reporting 2–hour postload glucose as the outcome. Begg's (p = 0.006) and Egger's (p < 0.001).

Table S2. Publication bias assessment for the 2-h postload glucose outcome.

Test Name	value	p
Fail-Safe N Calculation Using the Rosenthal Approach	213	<.001
Begg and Mazumdar Rank Correlation	0.500	.006
Egger's Regression	3.616	<.001
Trim and Fill Number of Studies	7	.
Corrected pooled mean difference (95% CI)	2.61 (0.43, 4.79)	0.019

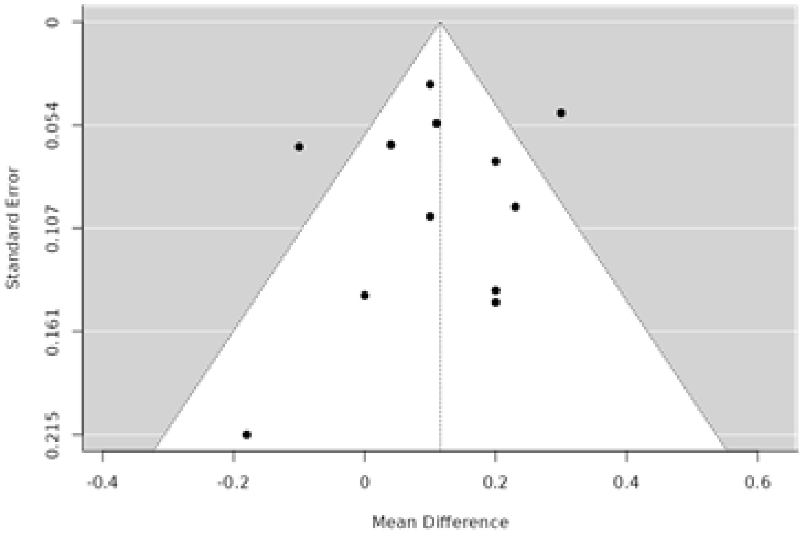

Figure S6. Funnel plot bias assessment of overall studies reporting A1c as the outcome. Begg's (p = 0.638) and Egger's (p = 0.539).

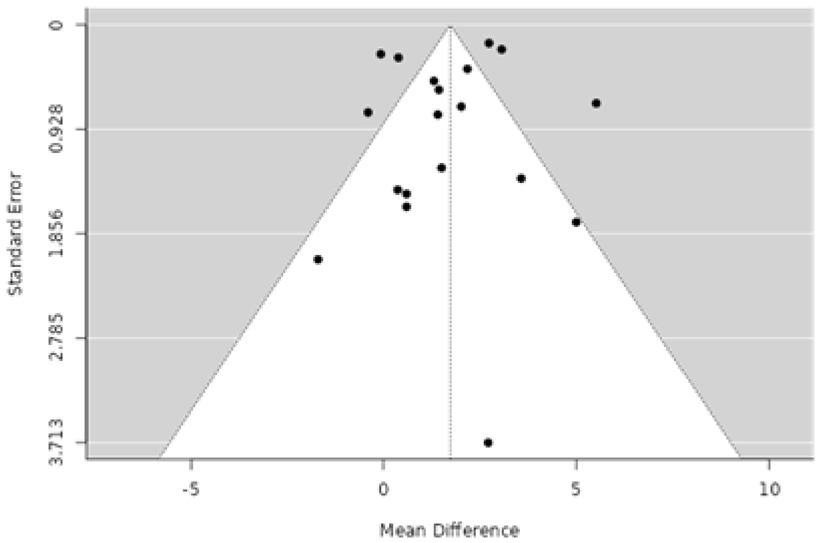

Figure S7. Funnel plot bias assessment of overall studies reporting fasting insulin as the outcome. Begg's (p = 0.945) and Egger's (p = 0.839).

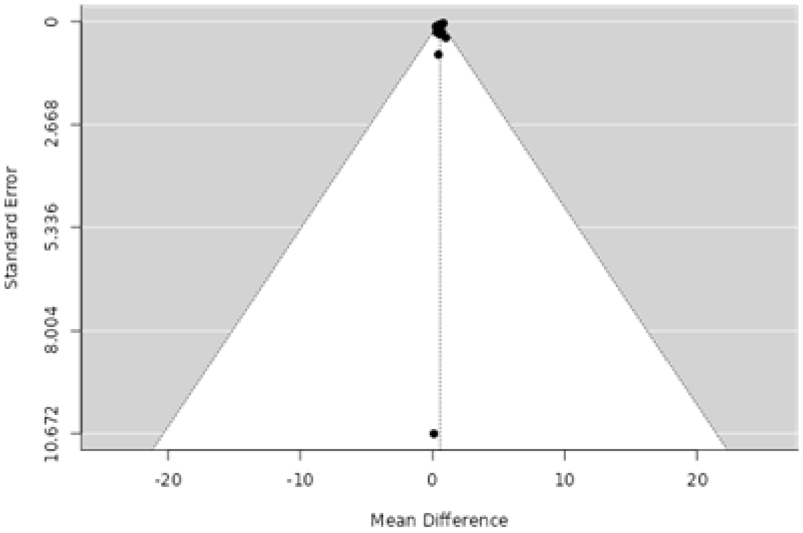

Figure S8. Funnel plot bias assessment of overall studies reporting HOMA as the outcome. Begg's (p = 0.590) and Egger's (p = 0.656).

Table S3. Certainty of evidence according to the GRADE approach.

Outcome	Nº studies (n)	Study design	Risk of bias	Inconsistency	Indirectness	Imprecision	Publication bias	Certainty
**fasting glucose**	26 (3122)	Observational	not serious	serious	not serious	not serious	undetected	⊕◯◯◯ Very low
**2-h postload glucose**	16 (2392)	Observational	not serious	not serious	not serious	not serious	suspected	⊕◯◯◯ Very low
**A1c**	12 (896)	Observational	not serious	serious	not serious	not serious	undetected	⊕◯◯◯ Very low
**fasting insulin**	19 (1452)	Observational	not serious	serious	not serious	not serious	undetected	⊕◯◯◯ Very low
**HOMA–IR**	13 (2428)	Observational	not serious	serious	not serious	not serious	undetected	⊕◯◯◯ Very low
